# OUTDOOR ACTIVITY ENGAGEMENT PRIOR TO AND DURING THE COVID-19 PANDEMIC IN SENIOR LIVING COMMUNITIES

**DOI:** 10.1093/geroni/igad104.1367

**Published:** 2023-12-21

**Authors:** Justine Sefcik, Elizabeth Euiler, Annalisa Na, Sherry Greenberg, Danielle Katsev, Rose Ann DiMaria-Ghalili

**Affiliations:** Drexel University College of Nursing and Health Professions, Philadelphia, Pennsylvania, United States; Drexel University College of Nursing and Health Professions, Philadelphia, Pennsylvania, United States; Drexel University, Philadelphia, Pennsylvania, United States; Monmouth University, Marjorie K. Unterberg School of Nursing & Health Studies, West Long Branch, New Jersey, United States; Drexel Univeresity, Philadelphia, Pennsylvania, United States; College of Nursing and Health Professions, Drexel University, Philadelphia, Pennsylvania, United States

## Abstract

There is growing evidence regarding the benefits of spending time outdoors. However, it is less understood if the COVID-19 pandemic has deterred or increased older adults’ outdoor leisure activities when they reside in senior living communities. This study aimed to discover how the pandemic affects senior living community-residing older adults’ outdoor activities. Older adults 65 and older were recruited through ResearchMatch and university partnerships. A qualitative descriptive approach was taken with semi-structured individual interviews conducted over Microsoft Teams, telephone, or in-person. A conventional content analysis was employed. Interviews were conducted in 2021-2022 with 4 females and 3 males from suburban Pennsylvania. While there were indoor restrictions and regulations (e.g., mask wearing when around others), the outdoor environments were less regulated and routine outdoor activities continued during the pandemic. Facilitators to outdoor activities were: 1) senior living campus features (e.g., paved walking paths, outdoor recreational sport areas), 2) structured outdoor activities (e.g., bocce ball and golfing tournaments facilitated by staff), and 3) access to transportation (e.g., to drive or visit other locations). A barrier to outdoor activities was: needing assistance from staff to exit buildings due to decreased mobility. This study illuminates that amenities and structured activities at senior living facilities promoted mobility in outdoor activities for those who preferred going outside. Findings inform design features that encourage mobility and outdoor activities among older adults. Policy implications include supporting senior living communities with less resources for maintaining campuses and neighborhoods, which could contribute to promoting outdoor activities and health equity.

